# Forty years of increasing suicide mortality in Poland: Undercounting amidst a hanging epidemic?

**DOI:** 10.1186/1471-2458-12-644

**Published:** 2012-08-11

**Authors:** Peter Höfer, Ian R H Rockett, Peeter Värnik, Elmar Etzersdorfer, Nestor D Kapusta

**Affiliations:** 1 Department of Psychiatry and Psychotherapy, Medical University of Vienna, Vienna, Austria; 2 Injury Control Research Center and Department of Epidemiology, West Virginia University, West Virginia, USA; 3 Estonian-Swedish Mental Health and Suicidology Institute, Estonian Centre of Behavioural and Health Sciences, and Tallinn University, Tallinn, Estonia; 4 Furtbach Hospital for Psychiatry and Psychotherapy, Stuttgart, Germany; 5 Department of Psychoanalysis and Psychotherapy, Medical University of Vienna, Währinger Gürtel 18-20, A-1090, Vienna, Austria

**Keywords:** Poland, Suicides, Validity, Misclassification, Reliability, Undetermined Intent

## Abstract

**Background:**

Suicide rate trends for Poland, one of the most populous countries in Europe, are not well documented. Moreover, the quality of the official Polish suicide statistics is unknown and requires in-depth investigation.

**Methods:**

Population and mortality data disaggregated by sex, age, manner, and cause were obtained from the Polish Central Statistics Office for the period 1970-2009. Suicides and deaths categorized as ‘undetermined injury intent,’ ‘unknown causes,’ and ‘unintentional poisonings’ were analyzed to estimate the reliability and sensitivity of suicide certification in Poland over three periods covered by ICD-8, ICD-9 and ICD-10, respectively. Time trends were assessed by the Spearman test for trend.

**Results:**

The official suicide rate increased by 51.3% in Poland between 1970 and 2009. There was an increasing excess suicide rate for males, culminating in a male-to-female ratio of 7:1. The dominant method, hanging, comprised 90% of all suicides by 2009. Factoring in deaths of undetermined intent only, estimated sensitivity of suicide certification was 77% overall, but lower for females than males. Not increasing linearly with age, the suicide rate peaked at ages 40-54 years.

**Conclusion:**

The suicide rate is increasing in Poland, which calls for a national prevention initiative. Hangings are the predominant suicide method based on official registration. However, suicide among females appears grossly underestimated given their lower estimated sensitivity of suicide certification, greater use of “soft” suicide methods, and the very high 7:1 male-to-female rate ratio. Changes in the ICD classification system resulted in a temporary suicide data blackout in 1980-1982, and significant modifications of the death categories of senility and unknown causes, after 1997, suggest the need for data quality surveillance.

## Background

In many countries of the European Union, suicide mortality exceeds that due to traffic accidents [1]. Suicides result from a complex interplay between mental health conditions, such as affective or substance abuse disorders, and socioeconomic factors [2,3]. Additionally, changes in political regimes, especially in former communist Eastern Europe, appear to have influenced the course of suicide rates [4,5]. Suicide and some other health-related statistics were kept secret in communist countries until 1988. However, despite publication, their validity remains questionable. While studies affirmed the reliability of suicide statistics in Russia, Ukraine, Belarus, Baltic and other countries [6,7], neither the reliability nor the validity of Polish data has been examined.

The validity of suicide certification covers two elements, specificity and sensitivity. Specificity measures false-negativity and sensitivity true positivity. Sensitivity seems the much more problematic of the two in democratic countries at least [8]. Alternatively stated, suicide undercounting is typically a much greater cause for scientific and public health concern than overcounting. Suicides are known to be hidden in other cause-of-death categories, predominantly ‘undetermined injury intent’ and ‘symptoms, signs, ill-defined conditions and unknown causes.’ Poisoning suicides, for example, seem highly prone to misclassification in other cause-of-death categories [9-11], especially ‘unintentional poisoning’ [12]. In general, death ascertainment procedures, such as performance of clinical and forensic autopsies, plausibly influence the sensitivity of suicide certification [13]. Thus, assessing the sensitivity as well as reliability of this certification is essential for comprehending the temporal course of suicide rates. Indeed, any evaluations of the impact of social, political, and economic change or interventions on suicide rates remain of unknown value in the absence of evidence on data quality.

No detailed analyses of Polish suicide statistics by method of suicide have previously been published. In addressing a research gap, we first documented the trend in Polish suicide rates from 1970 to 2009 by sex and method. We then estimated the reliability and sensitivity of the Polish suicide data to determine whether we could affirm their soundness [4]. Assessing data reliability was an imperative, since cause-of-death coding during our observation period involved three revisions of the *International Classification of Diseases* (ICD-8, -9 and -10), which might have impacted the reliability of the official suicide statistics.

## Methods

Population and mortality data, disaggregated by sex, age, manner, and cause, were obtained from the Polish Central Statistics Office for the period 1970-2009. Suicides in Poland were coded under ICD-8 and ICD-9 codes (E950-E959) for the periods 1970-1979 and 1980-1997, respectively, and under ICD-10 (X60-X84) for the period 1998-2009. Recorded on a death certificate, cause of death has to be determined by an authorized physician following an external body examination. While an autopsy can be requested by the physician prior to completion of a final death certificate in equivocal cases in Poland [14], the prevalence of autopsies is unknown [15].

To assess the temporal reliability and sensitivity of official Polish suicide statistics, we incorporated two causes of death that have been traditionally viewed in the literature as highly susceptible to hiding suicides, namely ‘undetermined injury intent’ (referred to as ‘undetermined intent’) and ‘ill-defined conditions and unknown causes’ (subsequently referred to as ‘unknown causes’) [8]. ‘Undetermined intent’ deaths were coded within E980-E989 under ICD-8 and ICD-9, and Y10-Y34 under ICD-10. Deaths from ‘unknown causes’ were coded under ICD-8 and ICD-9 as ‘E799 - Other ill-defined and unknown causes of morbidity and mortality,’ and under ICD-10 as ‘R99 – Other ill-defined and unknown causes of mortality’. ‘E797 - Senility without mention of psychosis’ (ICD-9 and ICD-10), and ‘R54 – Senility’ (ICD-10) were categorized under ‘unknown causes.’ In our reliability and sensitivity estimates, we also incorporated ‘unintentional poisoning’ deaths. These deaths were coded as E850-E869 (ICD-8 and ICD-9) or X40-49 (ICD-10).

To quantify the temporal reliability of Polish suicide data, we used the Spearman rank-order correlation coefficient to calculate the association between suicide rates and potential suicide reservoirs over time. A positive and high correlation indicated high data reliability. We estimated the sensitivity of Polish suicide certification, using a previously suggested procedure, which involved calculation of both upper and lower limits under alternative assumptions that deaths categorized as undetermined intent, unknown causes and/or unintentional poisonings, variously contained misclassified suicides [8]. Upper limits were calculated as the ratio of the suicide rate to the combined mortality rates for suicide and undetermined intent. The denominator in the lower limit estimates also incorporated respective mortality rates for unknown causes and unintentional poisonings.

Crude mortality rates were calculated as the annual number of deaths per 100,000 inhabitants, based on the corresponding average annual population. Time trends were estimated by the Spearman test for trend using the IBM SPSS Statistic 19.0 software package (SPSS Inc, Chicago, Illinois), and p-values were considered significant at the 0.05 level.

## Results

### Suicide rate changes, 1970-2009

**Figure 1:**
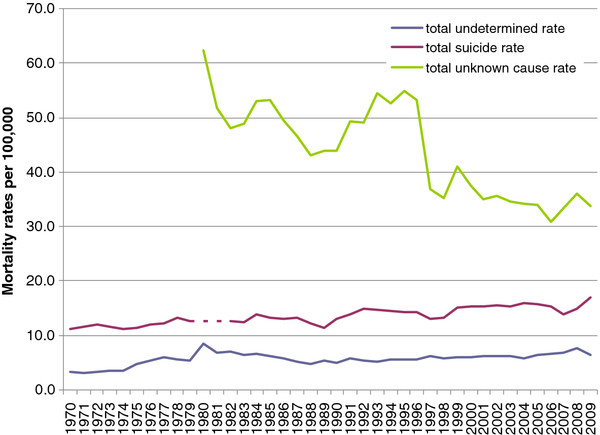
Total suicide, undetermined injury intent, and unknown cause death rates, Poland, 1970-2009.

**Figure 2:**
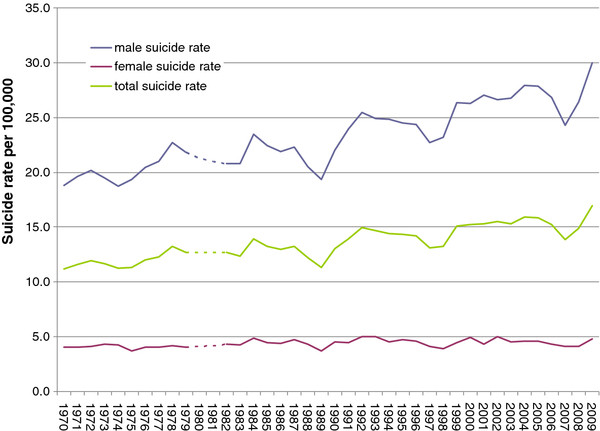
Suicide rates for Poland, 1970-2009 (dotted period: unreliable data).

During the period 1970-2009, a total of 190,559 deaths were registered as suicide in Poland. The suicide rate increased from 11.2 (1970) to 17.0 per 100,000 in 2009 (+51.3%). During the period of ICD-8 classification (1970-1979), the rate averaged 11.9 per 100,000 (SD ± 0.7), then increased to 13.5 (SD ± 1.1) during the ICD-9 period (1983 to 1996) and 15.0 (SD ± 1.1) during the period covered by ICD-10 (1997-2009). A change from ICD-8 to ICD-9 resulted in a temporary lack of suicide data for years 1980-1982 (Figures 1 and 2). The male suicide rate increased from 23.1 per 100,000 in 1970 to 35.1 in 2009. By contrast, the female suicide rate remained quite stable at 4.0 per 100,000 in 1970 and 4.8 in 2009. The net effect was an increase in the male-to-female suicide rate ratio from 5.7 in 1970 to 7.3 in 2009.

Age-specific suicide rates changed over the observation period. Rates for males aged 0-9 years (r = -0.65, p < 0.05) and 10-14 years (r = -0.55, p < 0.05) declined significantly. All other age groups showed significant increasing time trends (r = 0.38 to 0.81 at p < 0.05), except ages 15-19, which showed no change. Among females, suicide rates at ages 10-14 increased significantly (r = 0.38, p = 0.02), with declines observed at ages 15-19 (r = -0.52, p < 0.01) and 20-34 (r = -0.80, p < 0.01). No significant time trends manifested for remaining age groups.

The suicide rate for hanging, the most common suicide method regardless of sex, increased over the observation period from 8.8 to 15.2 per 100,000 (r = 0.92, p < 0.01). This corresponded to an increase in the prevalence of hangings among all suicides from 78.4% to 89.9%. Increase was observable for females (r = 0.80, p < 0.01) and males (r = 0.92, p < 0.01). The prevalence of suicide by poisoning, the second most common method for both sexes, declined from 10.6% in 1970 to 4.8% in 2009 (r = -0.87, p < 0.01). Decline was stronger among females (21.9 to 8.4%; r = -0.91, p < 0.01) than males (7.6 and 4.1%; r = -0.81, p < 0.01). Between 1970 and 2009, shooting suicide rates decreased for both females (r = -0.41, p < 0.01) and males (r = -0.50, p < 0.01), as did the respective drowning suicide rates (r = -0.74, p < 0.01) (r = 0.91, p < 0.01). On the other hand, the rate of suicide by jumping from a height increased for females (r = 0.46, p < 0.01) and males (r = 0.54, p < 0.01).

### Changing mortality categories implicating misclassification

**Figure 3:**
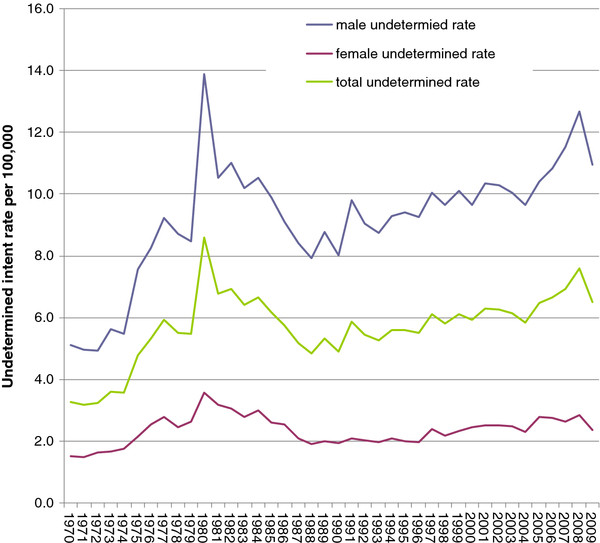
Undetermined intent death rates, Poland, 1970-2009.

An annual average 2,107 ±471 decedents had their cause-of-death classified as undetermined intent and 20,279 ±2,119 under unknown causes over the observation period. The total undetermined intent mortality rate increased from 3.3 per 100,000 in 1970 to 6.5 in 2009 (r = 0.74, p < 0.01). An increase was observed for both males and females. In 1980-1982, there was a marked peak in the rate of deaths of undetermined intent for both sexes, concomitant with the blackout on suicide data (Figure 3).

**Figure 4:**
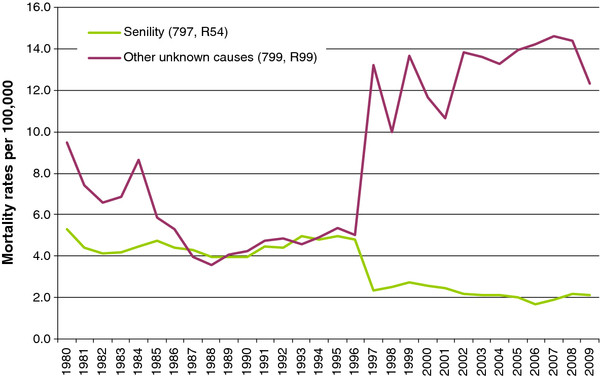
Ill-defined and unknown cause death rates, Poland, 1980-2009.

Data on unknown cause mortality (senility and other ill-defined deaths) were available only for the period 1980-2009. The total mortality rate for unknown causes averaged 50.4 ±4.8 deaths per 100,000 annually during 1980-1996, but significantly declined to 35.2 ±2.4 in the period 1997-2009 (r = -0.78, p < 0.01). However, this change from ICD-9 to ICD-10 showed an opposite impact on the respective mortality rates of senility and other ill-defined causes (Figure 4).

**Figure 5:**
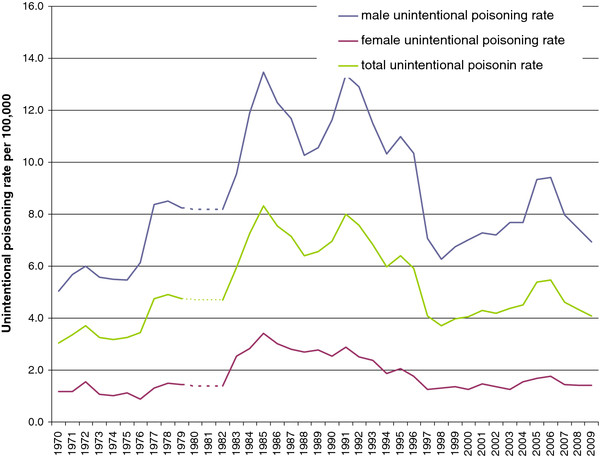
Unintentional poisoning death rates, Poland, 1970-2009.

A total of 92,397 unintentional poisoning deaths was registered in Poland between 1970 and 2009. The unintentional poisoning mortality rate increased from 3.0 per 100,000 in 1970 to 8.3 in 1985, before declining to 4.1 in 2009. This rate peaked for both males and females in 1985 (Figure 5). The Spearman rank-order correlation coefficient was r = 0.84 (p < 0.001) for the association between the suicide rates and the combined rates for suicide and death by undetermined intent. This correlation indicated high reliability for Polish suicide data. Reliability estimates were much higher for males (r = 0.97, p < 0.01) than females (r = 0.55, p < 0.01).

Table 1 presents mortality rates and estimated upper and lower limits of the sensitivity of suicide statistics for the periods subject to ICD-8, ICD-9, and ICD-10 classification, respectively. Upper limits ranged between 70% and 73%, and estimates were lower for females (64-68%) than males (71-75%). This sex differential was larger in the lower limit estimates, further indicating greater undercounting of female suicides.

**Table 1:** Mortality rates for selected causes, and sensitivity estimates for suicide certification by type of classification, Poland, 1970-2009

	**ICD-8**			**ICD-9**			**ICD-10**		
Time period	1970-1979			1983-1996			1997-2009		
**Rate per 100,000**	**Total**	**Males**	**Females**	**Total**	**Males**	**Females**	**Total**	**Males**	**Females**
Suicide	11.9	20.2	4.1	13.5	22.9	4.6	15.0	26.3	4.5
Undetermined intent	4.4	6.8	2.1	5.6	9.2	2.2	6.4	10.5	2.5
Unknown cause	-	-	-	49.7	37.2	61.5	35.2	31.6	38.6
Unintentional poisoning	3.8	6.5	1.2	6.9	11.5	2.6	4.4	7.5	1.4
**Sensitivity (%)**									
Upper limit ^1^	73	75	66	71	71	68	70	71	64
Lower limit ^2^	-	-	-	18	28	06	25	35	10

^1^ (suicides/suicides + deaths of undetermined intent) x 100.

^2^ (suicides/suicides + deaths of undetermined intent, unknown causes, and unintentional poisoning) x 100.

### Cross-sectional analysis, 2000-2009

In the final decade under review, there were 58,810 registered suicides in Poland, a suicide rate of 15.4 per 100,000. Hanging was the most common suicide method. This was the means employed in 90% of all suicides, 92% of male suicides, and 79% of female suicides. Among males, the second most common method was poisoning (2.4%), which was followed by jumping (1.9%) and shooting (1.0%). Among females, poisoning ranked second (8.4%) and jumping third (6.2%). Drowning was more common among females (2.7%) than males (0.4%). Female shooting suicides were negligible (<0.1%), and remaining methods accounted for 2.6% of male suicides and 3.8% of female suicides.

**Figure 6:**
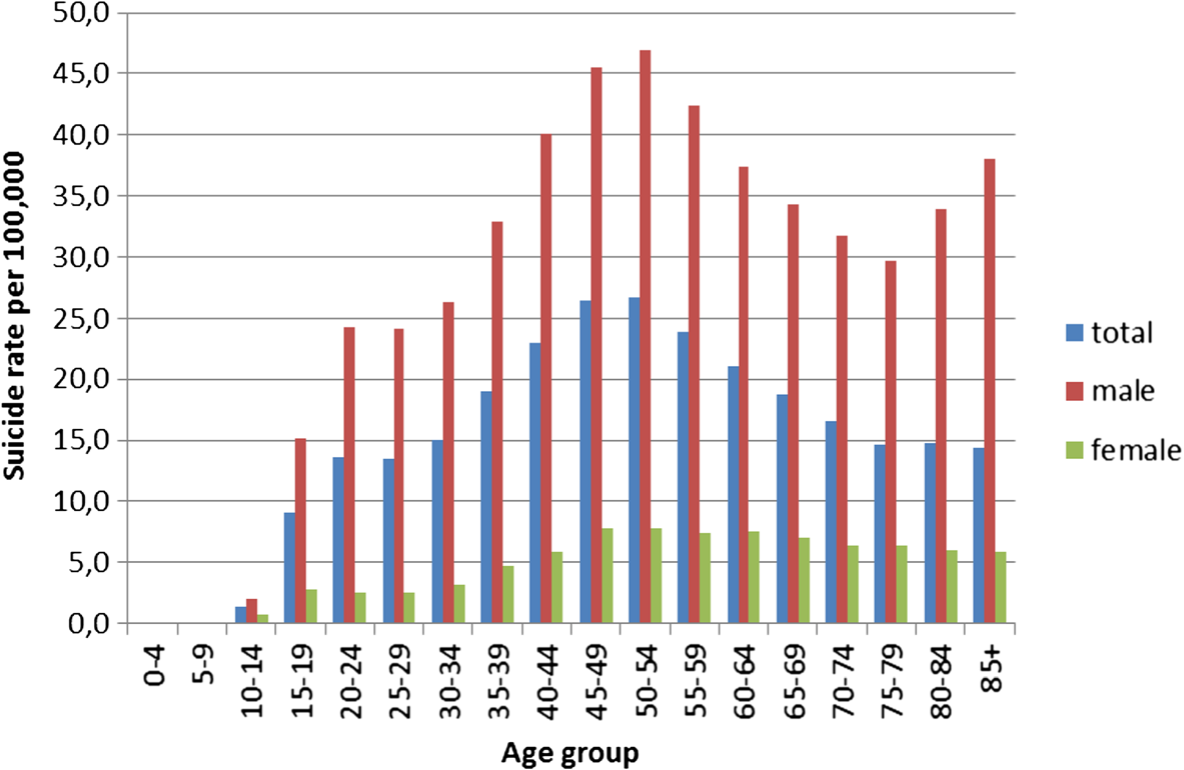
Annual average suicide rates by age and sex, Poland, 2000-2009.

The suicide rate peaked at ages 40-54 years (Figure 6). This middle-aged peak was much more pronounced for males than females. The suicide rate was quite low for the 75 years and older age group, and approximated the rate at ages 20-34.

## Discussion

Our study represents a comprehensive analysis of Polish suicide mortality statistics and their quality for the period 1970-2009. Besides the increasing suicide rate, a key finding was the high prevalence of hanging suicides and the relative peak in suicide rates in the age group of 40-54-year- olds. We also found that changes in the prevailing ICD classification system coincided with suicide mortality data deficits, an increase in the rate of deaths of undetermined intent, as well as major increases in the respective mortality rates for senility and unknown causes.

The suicide mortality rate in Poland is high according to global comparisons [16]. Even in Czarist “European Russia,” the areas that are now Polish territory had a high rate [4]. During our observation period, 1970-2009, the suicide rate in Poland rose substantially, a 51.3% increase. This large increase contrasted with the course of suicide rates in most other European countries [17]. These countries mainly showed rate declines that dated from the mid-1980s or early 1990s. Exceptions were Spain and Ireland, which also exhibited rate increases [18].

While the male suicide rate in Poland increased, the female rate remained quite stable over the 40-year observation period. The net result was an increasing male-to-female suicide rate ratio, which by 2009 had ascended to 7:1. This ratio is the highest in Europe, and one of the highest recorded in the world [19]. However, this magnitude may be artifactually high because of greater undercounting of female than male suicides. A competing explanation for the proportionally high male suicide rate could be reflected in the lower help-seeking behavior and consultation rates for males [20]. On the other hand, the “male depressive syndrome,” which is characterized by low stress tolerance, low impulse control, and antisocial behavior, and is strongly linked to suicide [21], may be grossly under diagnosed in Poland. In addition, the rapid social change in post-communist countries has been postulated to increase male vulnerability towards psychiatric disorders [22].

Turning to methods, hanging featured in almost 90% of all Polish suicides by the end of the observation period. Although hanging is a common method of suicide in Europe, its prevalence in Poland is the highest among European countries. Estonia has a lower prevalence of hanging suicides than Poland, and is differential by sex, 79% for males and 71% for females [23]. A lower prevalence with sex differentials were also reported for Germany (55.8% for males and 39.9% for females) [24], Austria (47.5% for males and 23.5% for females) [25], and Finland (37% for males and 26% for females) [26]. Unknown is whether the exceedingly high prevalence of hanging suicides in Poland is a genuine pattern or at least, in part, an artifact of serious underestimation of “soft” suicide methods, such as poisoning and drowning. This would also explain the high male-to-female suicide rate ratio, under the assumption that females more often use a “soft” method, such as poisoning [8]. On the other hand, an increasing prevalence of hanging suicides has also been reported for England over three decades [27], and in Australia and New Zealand, especially among young males [28,29].

The second most common suicide method among both sexes is poisoning, which showed a strong decline among Polish females during the observation period. The total poisoning rate for Poland conforms to the European average [23]. Relatively lower poisoning mortality rates were observed in Ireland and Estonia, which also have a high prevalence of hanging suicides. The rate of poisoning suicides also showed a considerable decline in Austria [25]. Until the mid-1980s, a poisoning suicide rate decline in Poland could have been offset by an increase in the unintentional poisoning mortality rate. However, both rates subsequently declined, which suggested a general decrease in poisoning death registrations irrespective of decedent intent. Nevertheless, although estimates of Polish autopsy rates are unavailable, a likely decrease in both autopsies and toxicological analyses could have art factually contributed to the decrease in registrations [13].

Increased suicide rates were found among adolescent females under age 14 years, and in young males aged 20 years and older. Using an overlapping category, males aged 15-29 manifested increasing suicide rates in Poland, while showing rate declines in other EU countries [30]. An upward trend in the suicide rate among children and young adolescents, up to age 14, has been observed in many countries, and in contrast to the Polish data, especially in the case of males [31]. Nevertheless, large cross-national differences are apparent. For example, suicide rates are increasing among youth in the United States, stable in the United Kingdom, and decreasing in Austria [32-34] (McClure et al. 2001, CDC 2004, Dervic et al. 2006). Reasons for the increasing suicide rates among very young Polish females remain unknown, a research gap that argues for in-depth investigations.

Suicide rates in Poland do not increase with age like the “Hungarian” pattern reported for most European countries, as, for example, Hungary, Germany, and Austria [25,35]. Polish suicide rates peak at ages 40-54, and then decrease across older age groups before rising again at ages 80 years and older. Typifying Eastern Europe, this pattern is pronounced in the former Soviet countries. The peak at middle age is paralleled in the United States in the case of female suicides [12]. Although an empirical question, the overall "Camel" pattern, which we observed for age-specific suicide rates in Poland, likely variously implicates the combination of prevailing classification procedures and social, cultural, and economic conditions.

The prevalence of non-hanging suicides in Poland is very low. This finding alone suggests that poisonings and other “soft” suicide methods might be underrepresented in official Polish suicide statistics because of misclassification [8]. However, the high mortality rates for undetermined intent and unknown causes, respectively, point to an even larger potential reservoir for misclassified suicides. Adopting a previously suggested method for quantifying sensitivity of suicide certification [8], we generated upper-level estimates (percent of true-positives) of 70-73%. When we factored into our sensitivity calculations unknown causes and unintentional poisonings, as mortality categories also prone to masking suicides, the ensuing lower-level estimates indicated that the Polish suicide data were extremely deficient. Our results also indicate that suicide misclassification is even more problematic for females than males. This inference is consistent with the excessively high male-to-female suicide rate ratio and the large sex differential in the prevalence of non-hanging suicides. In short, “softer” suicide methods and female suicides both appear highly prone to misclassification in Poland.

The blackout on Polish suicide data for the years 1980-1982, following the change from ICD-8 to ICD-9 coding, coincided with a sharp increase in the rate of undetermined intent deaths. This finding strongly suggests that undetermined deaths might indeed have been a reservoir for suicides during that interval. However, Polish suicide data otherwise appear temporally reliable. While suicide data deficits also occurred during these years in four countries neighboring Poland, namely, Belarus, Lithuania, Ukraine, and Russia [36], they were not investigated in detail. A recent study more generally showed that changing cause-of-death classification procedures may mean a redistribution of deaths across external cause categories [37].

According to the International Association for Suicide Prevention (IASP), six major approaches have been proposed for preventing suicide: treatment of mental disorders, firearm control, detoxification of domestic gas and car emissions, control of toxic substances, and restrained media reporting [19]. Because of the excessively high hanging suicide rate in Poland, concerted efforts are needed for diagnosing and treating psychiatric disorders, including affective and alcohol or other substance abuse disorders. These efforts would necessarily be predicated on the assumption that careful assessment, diagnosis, and management of psychiatric conditions can reduce suicidal behavior.

## Conclusion

The high rate of increase in the Polish suicide rate calls for a national prevention initiative. Contrary to the overall trend of suicide rate reduction in both Eastern and Western Europe, the Polish suicide rate trended strongly upwards between 1970 and 2009. Estimated sensitivity of Polish suicide certification is very low relative to that of some other countries, such as Hungary and Austria [17,36,38]. In light of the low sensitivity estimates, an exceptionally high male-to-female suicide rate ratio, and a questionably low prevalence of suicide by “soft” methods, it seems highly plausible that suicides rates in Poland appear gross underestimates despite the large secular increase.

Changes in the ICD classification system produced a temporary suicide data blackout (1980-1982). This observation, in concert with significant increases in respective mortality rates for senility and unknown causes, after 1997, collectively support our recommendations for routine monitoring of Polish suicide data quality and exercise of caution in comparing suicide rates in Poland with those of other nations. We do conclude that Polish suicide statistics seem highly reliable across time. We acknowledge that our study only provides an indirect assessment of Polish suicide data quality. Nonetheless, the results expose the importance of in-depth investigations, which include psychological autopsies, on deaths classified under undetermined intent and senility or other unknown causes. Such investigations must be integral to the comprehensive approach that is needed in Poland to optimize suicide data quality assurance and associated suicide risk-factor delineation, risk-group identification, intervention, and evaluation.

## Competing interests

The authors declare that they have no competing interests.

## Authors' contributions

PH and NDK conceived and designed the study. PH and NDK obtained, prepared, and managed the data, performed the statistical analyses and conducted the literature review. PH, IRHR, PV, EE and NDK interpreted the findings, and drafted the manuscript. All authors read and approved the final manuscript.

## Pre-publication history

The pre-publication history for this paper can be accessed here:

http://www.biomedcentral.com/1471-2458/12/644/prepub
